# Complete Genome of an Alkali‐Resistant *Rhizobium anhuiense* Symbiont of Pea Reveals Species‐Specific Plasmid Fusion and Genomic Plasticity

**DOI:** 10.1111/1758-2229.70366

**Published:** 2026-05-25

**Authors:** Jiashun Miao, Chi Zhang, Qifan Jiang, Zhangliang Yao, Kuirong Cao, Jie Chen, Hua Wang, Na Liu

**Affiliations:** ^1^ Xianghu Laboratory Hangzhou China; ^2^ State Key Laboratory for Quality and Safety of Agro‐Products, Zhejiang Academy of Agricultural Sciences Hangzhou China; ^3^ Jiaxing Academy of Agricultural Sciences, Institute of Eco‐Environmental Jiaxing China

**Keywords:** megaplasmid fusion, *Pisum sativum*, plant growth‐promoting rhizobacteria, *Rhizobium anhuiense*, symbiotic nitrogen fixation

## Abstract

The rhizosphere microbiome is crucial for plant growth and stress resilience in sustainable horticulture. Here, we report the complete genome assembly and functional characterisation of *Rhizobium anhuiense* Xianghu001, a nitrogen‐fixing symbiont isolated from pea ( *Pisum sativum* ) root nodules. A hybrid assembly strategy combining PacBio reads and Illumina reads yielded a 7.36 Mb high‐quality assembly comprising one chromosome, one megaplasmid and four accessory plasmids, encoding 6899 protein‐coding genes, of which 66.64% are located on the chromosome. Phylogenomics and synteny confirmed its placement within *R. anhuiense*. We detected a lineage‐specific plasmid fusion forming the megaplasmid, while three accessory plasmids appear to be strain‐specific and potentially acquired via horizontal gene transfer. Insertion sequence profiling suggests genome rearrangement shaping plasmid structure. To explore intraspecies diversity, we sequenced six additional local *R. anhuiense* isolates from pea. Despite their close geographic origin, genomic comparison revealed extensive divergence. Phenotypic assays demonstrated that Xianghu001 significantly promotes pea growth under nitrogen‐deficient conditions, increasing chlorophyll content and nitrogen accumulation. It synthesises high levels of IAA (~184 mg/L), tolerates mild salinity (≤ 0.15% NaCl) and grows optimally at alkaline pH (8.0–10.0). Our findings provide a comprehensive genomic and functional framework for *R. anhuiense* Xianghu001 and underscore its potential as a biofertiliser.

## Graphical abstract

*Rhizobium anhuiense* strain Xianghu001 is an alkali‐tolerant, nitrogen‐fixing symbiont isolated from pea ( *Pisum sativum* ) root nodules. Genome sequencing revealed a lineage‐specific megaplasmid fusion and high plasmid plasticity. Functional assays confirmed its plant growth‐promoting traits, including increased total nitrogen content and IAA production. Population genetic and phylogenomic analyses highlighted its distinct evolutionary position and intra‐species diversity. These features underscore Xianghu001's potential as a robust biofertiliser for sustainable legume cultivation.

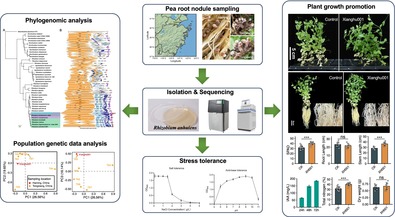

## 1 Introduction

Pea ( *Pisum sativum* L.) is one of the most widely cultivated legume crops globally, valued for its high nutritional content and biological nitrogen fixation capacity (Zahran 1999; Gage 2004; Herridge et al. 2008). It plays a significant role in green agriculture, crop rotation systems and sustainable agricultural development (Ćujić Nikolić et al. 2026). The symbiotic nitrogen‐fixing system formed between legumes and rhizobia is one of the most important biological nitrogen fixation mechanisms in nature (Udvardi and Poole 2013). During symbiosis with legumes, rhizobia invade the root hairs and enter the root cortex under the induction of plant‐specific signalling molecules, initiating the formation of root nodules. Within these nodules, rhizobia differentiate into bacteroids, which fix atmospheric nitrogen and convert it into ammonium, thereby meeting the nitrogen requirements of the host plant (Yang et al. 2022). This system helps reduce reliance on chemical nitrogen fertilisers, improves soil fertility, lowers agricultural production costs and mitigates environmental pollution (Bhatt et al. 2014).

Extensive genetic studies have been conducted on various rhizobial species. Some rhizobia can only have an exclusive symbiosis with one species, including *Sinorhizobium meliloti* (the symbiont of alfalfa), *Bradyrhizobium japonicum* (soybean) (Pellock et al. 2000; Capela et al. 2006; Zilli et al. 2011). And there are also some rhizobia with a relatively wide range of hosts, such as *Rhizobium leguminosarum* , which can be symbiotic with different legumes such as peas, broad beans (biovar *viciae*), clovers (biovar *trifolii*) and kidney beans (biovar *phaseoli*) (Young et al. 2006; Andronov et al. 2015). On the other hand, pea can also form symbiotic relationships with a wide variety of rhizobia species in addition to *R. leguminosarum* bv. *viciae*, indicating a broader symbiont diversity than previously recognised (El‐Hamdaoui et al. 2003; Ilahi et al. 2021).

Although some commercial rhizobial inoculants for peas are already available, their application in diverse ecological regions remains limited due to constraints such as narrow genetic diversity, poor environmental adaptability, inconsistent nitrogen fixation efficiency and incompatibility with certain pea varieties (O'Callaghan et al. 2022). In contrast, some high‐efficiency rhizobial strains exhibit strong compatibility with specific cultivars, significantly enhancing nodulation, nitrogen fixation and crop yield (Laguerre et al. 2007; Mendoza‐Suárez et al. 2021). Therefore, the isolation and identification of novel *Rhizobium* strains with high nitrogen fixation capacity, broad adaptability and strong compatibility with peas hold great research significance and promising application prospects in modern agricultural production.

Among rhizobia, members of the genus *Rhizobium* establish symbiotic relationships with a wide range of leguminous hosts, forming specialised structures known as root nodules. *Rhizobium anhuiense*, first described as a symbiont of various leguminous crops, is increasingly recognised for its ecological importance and potential application in sustainable agriculture (Travin et al. 2022).

Genomic investigations of rhizobia have revealed that symbiotic and accessory functions are often associated with plasmids or megaplasmids, whose structural evolution—including fusion, fission and horizontal gene transfer events—can profoundly influence host specificity, symbiotic efficiency and environmental adaptability (Finan 2002; Andrews et al. 2018). Despite its agricultural relevance, the genome organisation and plasmid dynamics within *R. anhuiense* populations remain poorly understood. In particular, the evolutionary history and genetic diversity of strains inhabiting geographically proximate but distinct environments have yet to be thoroughly explored.

In this study, we generated a complete genome assembly for the *R. anhuiense* strain Xianghu001, isolated from pea root nodules in Haining City, Zhejiang Province, China, using a combination of long‐read and short‐read sequencing technologies. Comparative genomic analyses revealed a species‐specific megaplasmid fusion event in *R. anhuiense*, as well as the acquisition of strain‐specific plasmids likely originating through horizontal gene transfer. Furthermore, by analysing additional isolates from nearby Tongxiang, we uncovered unexpectedly high levels of intra‐species genetic diversity, with significant morphological and genomic divergence among *R. anhuiense* strains collected from locations less than 10 km apart. These findings provide new insights into the genome evolution, plasmid dynamics and population structure of *R. anhuiense*, expanding our understanding of rhizobial diversity and adaptation.

## 2 Materials and Methods

### 2.1 Isolation of the Strains and Phenotypic Characterisation

Strain Xianghu001 was isolated from root nodules of field pea ( *Pisum sativum* L. cv. Zhewan No. 1) grown in Haining City, Zhejiang Province, China (30.280055° N, 120.181901° E). Root nodules were first rinsed under running water and then surface‐sterilised by sequential immersion in 70% ethanol and 2% sodium hypochlorite for 1 min each. After sterilisation, the nodules were crushed and the suspension was streaked onto yeast mannitol agar (YMA) plates, followed by incubation at 28°C for 48 h. Individual colonies were subsequently subcultured on fresh YMA plates to obtain pure cultures. The isolates were preserved in 60% glycerol at −80°C for long‐term storage. In addition, six strains (designated with the prefix ‘TX’) were obtained from root nodules of field pea ( *P. sativum* L.) collected from the Family Farm of Zhangjidong in Tongxiang City, Zhejiang Province, China (30.291577° N, 120.244434° E) using the same protocol.

To assess salt tolerance, yeast mannitol broth (YMB) was supplemented with NaCl at final concentrations of 0.1, 0.5, 1.0, 1.5, 2.0, 3.0, 4.0 and 5.0 g/L. To evaluate pH tolerance, YMB media were adjusted to pH values ranging from 4.0 to 11.0 (in increments of 1.0) using appropriate buffer systems. All cultures were incubated at 28°C with shaking at 200 rpm for 48 h. Bacterial growth was quantified by measuring the optical density at 600 nm (OD_600_). Specifically, the growth of strains was categorised according to the optical density at 600 nm (OD600) measured after incubation: positive growth (+, OD_600_ ≥ 1.0), weak growth (w, 0.1 ≤ OD_600_ < 1.0) and negative growth (−, OD_600_ < 0.1).

### 2.2 Indole‐3‐Acetic Acid (IAA) Quantification

IAA production by *Rhizobium anhuiense* was determined using a spectrophotometric assay with Salkowski reagent (Guardado‐Fierros et al. 2024). IAA standard solutions (20, 50, 100, 150 and 200 mg/L) were prepared and mixed in equal volume with Salkowski reagent (50 mL of 35% HClO_4_ containing 1 mL of 0.5 M FeCl_3_). The mixtures were incubated in the dark at room temperature for 30 min and absorbance was measured at 535 nm to generate a standard calibration curve.

*R. anhuiense* were activated and cultured in YEM liquid medium (KH_2_PO_4_ 0.25 g/L, K_2_HPO_4_ 0.25 g/L, MgSO_4_·7H_2_O 0.2 g/L, NaCl 0.1 g/L, yeast extract 0.8 g/L, mannitol 10.0 g/L, agar 18.0 g/L, pH 7.2) supplemented with 0.5 g/L tryptophan at 30°C with shaking at 140 rpm for 24, 48 and 72 h. At each time point, 2 mL of culture broth was centrifuged at 10,000 rpm for 5 min. Then, 1 mL of the resulting supernatant was mixed with 1 mL of Salkowski reagent and incubated in the dark for 30 min at room temperature. Absorbance was read at 535 nm and IAA concentration was calculated based on the standard curve.

### 2.3 Plant Culture and Rhizobial Inoculation

Pea seeds ( *Pisum sativum* L. cultivar ZW No. 1) were germinated in vermiculite and grown in a controlled growth chamber at 28°C under a 16/8 h light/dark photoperiod for 4 weeks (Liu et al. 2024). *R. anhuiense* strain Xianghu001 was activated and cultured in YEM medium to exponential growth phase (OD_600_≈0.5). The culture was then centrifuged, resuspended in sterile water and adjusted to OD_600_ = 0.1. For inoculation, 5 mL of the bacterial suspension was applied to each pea seedling. After treatment, phenotypic traits including shoot height, root length and SPAD chlorophyll index were measured in both inoculated and uninoculated control plants. Representative plants were photographed and whole‐plant samples were collected for total nitrogen content analysis.

### 2.4 Leaf Chlorophyll Estimation

Chlorophyll content in seedling leaves was measured using a handheld SPAD‐502 chlorophyll meter (Minolta Corp., Osaka, Japan). Measurements were taken at consistent positions on fully expanded, mature leaves, avoiding the central midvein. For each leaf, three SPAD readings were recorded and averaged to obtain the mean chlorophyll index.

### 2.5 Total Nitrogen Determination

Total nitrogen content in plant samples was determined using an elemental analyser (Elementar Vario EL III, Germany). Approximately 2–5 mg of dried and finely ground samples were accurately weighed using a microbalance (precision: 0.000001 g) and sealed in tin capsules for analysis. The instrument was calibrated with sulfanilamide standard (2–5 mg) and the calibration curve was established by plotting the peak area against nitrogen concentration (%). The instrument parameters were set as follows: combustion tube temperature 1150°C, reduction tube temperature 850°C and oxygen flow rate 40 mL/min. Other parameters were maintained at default settings. Nitrogen gas (N_2_) produced by high‐temperature catalytic combustion was separated by a chromatographic column and detected using a thermal conductivity detector. The nitrogen content was automatically calculated by the instrument software using the standard calibration curve.

### 2.6 Library Construction and Sequencing

To prevent the loss of small plasmid information (< 15 kb) that may occur when using PacBio libraries alone (typically with 10–20 kb insert sizes), a combination of PacBio long‐read sequencing and Illumina paired‐end short‐read (150 bp) sequencing was employed.

Genomic DNA for short‐read sequencing was extracted using bacterial DNA extraction kit (magnetic beads) (Majorbio, shanghai, China) according to manufacturer's protocol. Purified genomic DNA was sheared into ~450 bp fragments using a Covaris ultrasonicator. The fragmented DNA was then transferred to construct a sequencing library and sequenced on the Illumina platform in 2 × 150 bp paired‐end mode.

High‐molecular‐weight genomic DNA for long‐read sequencing was extracted using a standard phenol‐chloroform protocol. DNA quality and purity were evaluated using a NanoDrop spectrophotometer (A260/A280 = 1.8–2.0) and concentration was quantified using a TBS‐380 fluorometer. Only samples showing no degradation and with a total yield of ≥ 15 μg were selected for library construction. Genomic DNA was sheared into ~8–10 kb fragments using G‐Tubes (Covaris), followed by end‐repair to generate blunt‐ended fragments. Hairpin adapters were then ligated to both ends of the double‐stranded DNA to form circular SMRTbell templates. These dumbbell‐shaped constructs enable continuous circular sequencing of individual DNA molecules. The SMRTbell libraries were annealed to sequencing primers and bound to DNA polymerase using the appropriate binding kit. The resulting polymerase‐SMRTbell complexes were loaded into zero‐mode waveguides (ZMWs) on a SMRT Cell. Real‐time sequencing was performed using the PacBio Sequel II platform.

### 2.7 De Novo Assembly of Genome

Both raw paired‐end short reads generated by Illumina platform and long reads generated from PacBio Revio platform were fed to Unicycler (version: v0.4.8) for genome assembly (Wick et al. 2017). Unicycler uses the SPAdes (version 3.13.1) read error correction module to reduce the number of errors in the short read before SPAdes assembly (Wick et al. 2017). To rule out the bias generated by denovo assembly, Canu (version 2.2) (Koren et al. 2017) and Flye (Kolmogorov et al. 2019) (version 2.9.5‐b1801) were also employed to assemble the genome by only using the PacBio long reads with default parameters (Koren et al. 2017; Kolmogorov et al. 2019). Plasmids were identified using the PlasFlow software (https://github.com/smaegol/PlasFlow) (Krawczyk et al. 2018). The identified plasmid sequences were then annotated using BLAST (Camacho et al. 2009) searches against the PLSDB database (https://ccb‐microbe.cs.uni‐saarland.de/plsdb/) (Schmartz et al. 2022). The genome landscape was visualised using Circos (version 0.69‐9) (Krzywinski et al. 2009). Genome‐wide GC content profiles for Xianghu001 were calculated from the assembled genome using SeqKit (seqkit sliding and seqkit fx2tab) with non‐overlapping windows of 1 kb (Shen et al. 2016).

### 2.8 Genome Annotation and Synteny Analysis

RepeatMasker was used to predict interspersed repeats (Tarailo‐Graovac and Chen 2009). Tandem Repeats Finder was employed to predict tandem repeats in the genome assembly (Benson 1999). Prodigal is used by default to predict genes in chromosomal sequences, while GeneMarkS is used for plasmid genome prediction (Besemer 2001; Hyatt et al. 2010). tRNA genes were identified using tRNAscan‐SE v2.0 (http://trna.ucsc.edu/software/) (Lowe and Eddy 1997). rRNA genes are predicted using the Barrnap software (https://github.com/tseemann/barrnap). Prediction of potential small RNAs (sRNAs) was performed using the Infernal software (http://eddylab.org/infernal/) in conjunction with the Rfam database (https://rfam.xfam.org/)(Nawrocki and Eddy 2013). NR (Non‐Redundant Protein Database), Swissprot, Pfam, EggNOG and GO (Gene Ontology), KEGG (Kyoto Encyclopedia of Genes and Genomes) databases were use to perform functional annotation (Kanehisa and Goto 2000). The genome assemblies used in comparative genomics analysis were downloaded from NCBI GenBank/RefSeq databases and that their accession numbers are provided in Table S9. The genomic synteny among different genome assemblies was visualised by using GENESPACE (version 1.2.3) (https://github.com/jtlovell/GENESPACE). Pairwise genome alignments between different genome assemblies were performed using minimap2 (version 2.28‐r1245‐dirty) (Li 2018) and the resulting alignment coordinates were parsed and visualised using the ggplot2 package in R (version 4.4.1).

### 2.9 Genetic Diversity, Phylogenetics and Population Genetics Analysis

The raw paired‐end sequencing data of all strains generated from Illumina platform was firstly quality filtered by using fastp (version 0.20.1) with default parameters (Chen et al. 2018). The clean reads of each sample were then mapped to the genome assembly of strain Xianghu001 using bwa mem (version 0.7.17‐r1188) with default parameters (Li and Durbin 2010). The aligned reads were sorted by coordinate by using samtools sort function (version 1.13) and then the duplicated reads were removed using Picard (version 3.4.0) (Li et al. 2009). The read depth across the reference genome of each sample was calculated with the alignment files generated above using the software PanDepth (version 2.25) with 1 kb non‐overlap sliding window (Yu et al. 2024). Variant calling was performed using the Genome Analysis Toolkit (GATK v4.0.1) HaplotypeCaller in GVCF mode (McKenna et al. 2010). Individual GVCFs were jointly genotyped using GenotypeGVCFs with default parameters. Raw SNP variants were subjected to hard filtering using GATK VariantFiltration with the following criteria: QD < 2.0, FS > 60.0, MQ < 40.0, MQRankSum < −12.5 and ReadPosRankSum < −8.0; variants failing any threshold were removed. The resulting SNP dataset was used for downstream population genetic and phylogenetic analyses. Principal component analysis (PCA) was performed using GCTA (v1.94.1) (Yang et al. 2011). The first three principal components were extracted and visualised in R v4.3.2 using the ggplot2 package (Wickham 2016). Phylogenetic reconstruction was performed using IQ‐TREE2 (v2.2.2.6) (Nguyen et al. 2015). The best‐fit substitution model was selected using the built‐in ModelFinder according to the Bayesian Information Criterion (BIC). Branch support was assessed using 1000 ultrafast bootstrap replicates (UFBoot). The resulting phylogenetic trees were visualised and edited using FigTree v1.4.4. Mash distance between each sample pairs was calculated using the software Mash (version 2.3) (Ondov et al. 2016). Average Nucleotide Identity (ANI) between each sample pair was calculated using pyani (v0.3) with anim mode (https://github.com/widdowquinn/pyani).

## 3 Results

### 3.1 Complete Genome Assembly of *Rhizobium* Strain Xianghu001

**FIGURE 1:**
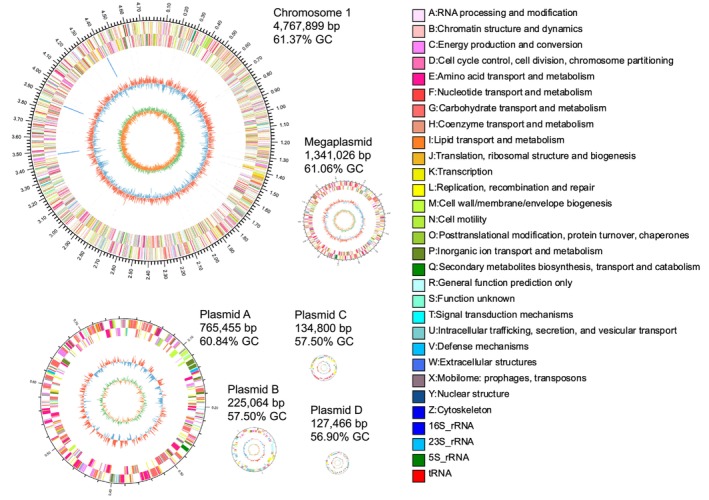
The chromosome, megaplasmid and four plasmids of strain Xianghu001. The four plasmids are shown at the same relative scale and the chromosome and megaplasmid at another same relative scale. The outermost ring of the circular map indicates genome size. The second and third rings represent coding sequences (CDSs) on the forward and reverse strands, respectively, with different colours corresponding to different COG functional categories. The fourth ring displays the positions of rRNA and tRNA genes. The fifth ring shows the GC content: Red outward peaks represent regions with GC content higher than the genome‐wide average, where taller peaks indicate greater deviations; blue inward peaks represent regions with lower GC content, with deeper troughs reflecting greater differences from the average. The innermost ring illustrates the GC skew, calculated as (G−C)/(G + C), which can help infer the leading and lagging strands. Typically, the GC skew is positive on the leading strand and negative on the lagging strand.

A total of 73,436 long reads, comprising 897,688,029 bp (121.94× coverage based on the final genome size), were generated using the PacBio Sequel II platform (Table S1). The read N50 was 12,832 bp (Table S2). Additionally, 1,335,762,308 bp of short reads were generated from the Illumina platform (Table S3). By combining both long and short reads, we assembled the genome of strain Xianghu001, resulting in a total genome size of 7,361,690 bp, which is slightly larger than that of *Rhizobium anhuiense* bv. *trifolii* (GCF_024346605.1; 7,327,117 bp) (Travin et al. 2022), but a little bit smaller than that of *R. leguminosarum* (7,751,309 bp) (Young et al. 2006). The assembly comprised one circular chromosome (4,767,899 bp), one circular megaplasmid (1,341,026 bp) and four circular plasmids (Figure 1). The plasmids were identified and annotated using PlasFlow and BLAST searches against the PLSDB database. The overall GC content of the genome is approximately 61.00%. The GC contents of the chromosome, megaplasmid, plasmid A, plasmid B, plasmid C and plasmid D are approximately 61.36%, 61.06%, 60.84%, 57.50%, 58.27% and 56.90%, respectively (Table S4). Genome completeness assessments indicated high assembly quality. BUSCO analysis showed 100% completeness and CheckM evaluation indicated 100% completeness with a 0.25% contamination rate and 0 strain heterogeneity (Tables S5 and S6). 100% of the genome of Xianghu001 has sequencing reads covered (Table S7).

A total of 4287 bp of interspersed repeats, accounting for 0.06% of the genome, were identified (Table S8). The genome encodes 6899 predicted protein‐coding genes, of which 66.64% (4638) are located on the chromosome. Protein‐coding regions span 6,389,067 bp, representing 86.79% of the genome assembly, with an average gene length of 926.09 bp. The GC content in gene regions (61.90%) is higher than that in intergenic regions (55.11%). Additionally, 52 tRNA genes representing 20 different tRNA types were identified. The genome also harbours 9 rRNA genes, comprising three copies each of 16S, 23S and 5S rRNAs. Furthermore, 83 sRNA genes were identified, totaling 9706 bp (0.13% of the genome). Functional annotation revealed that 6882 protein‐coding genes were annotated in NCBI nr, 5031 in Swiss‐Prot, 5890 in Pfam, 5110 in KEGG, 5777 in COG and 3559 in the Gene Ontology (GO) database.

### 3.2 Comparative Genomic Analysis Reveals *Rhizobium anhuiense*‐Specific Plasmid Fusion Events and Strain‐Specific Plasmid Acquisition

**FIGURE 2:**
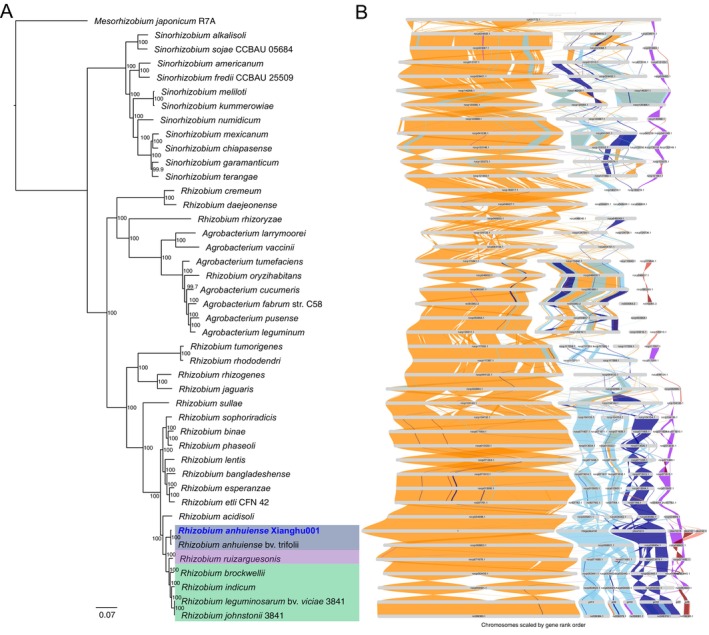
Phylogenetic relationships and chromosomal synteny among *Rhizobium* and related species. (A) Maximum likelihood phylogenetic tree based on whole genomic protein‐coding gene alignments of *Rhizobium*, *Agrobacterium*, *Sinorhizobium* and *Mesorhizobium* species. Bootstrap support values are indicated at each node. Strains closely related to *Rhizobium anhuiense* Xianghu001, including *Rhizobium anhuiense* bv. *trifolii*, *Rhizobium ruizarguesonis*, *Rhizobium brockwellii*, *Rhizobium indicum*, *Rhizobium leguminosarum* bv. *viciae* 3841 and *Rhizobium johnstonii* 3841, are highlighted in colour. Notably, strain *R. leguminosarum* bv. *viciae* 3841 was reclassified as *Rhizobium johnstonii* 3841 in 2023 (Young et al. 2023); thus, the two labels in the figures refer to the same strain and genome. (B) Chromosomal synteny analysis among Xianghu001 and representative *Rhizobium* strains. Chromosomes are scaled by gene rank order. Different coloured ribbons indicate syntenic relationships between genomes.

Phylogenetic analyses based on 16S rRNA genes, housekeeping genes and whole‐genome protein‐coding genes consistently supported that the strain isolated from *Pisum sativum* nodules in Haiyan, China belongs to *Rhizobium anhuiense* (Figure 2; Figures S1 and S2) (Zhang et al. 2015; Travin et al. 2022). Average Nucleotide Identity (ANI) analyses also confirmed that strain Xianghu001 belongs to *Rhizobium anhuiense* (Figure S3). *R. anhuiense* was first isolated from effective nodules of *Vicia faba* and *Pisum sativum* in 2015 in Anhui and Jiangxi Provinces of China and proposed as a novel species (Zhang et al. 2015).

**FIGURE 3:**
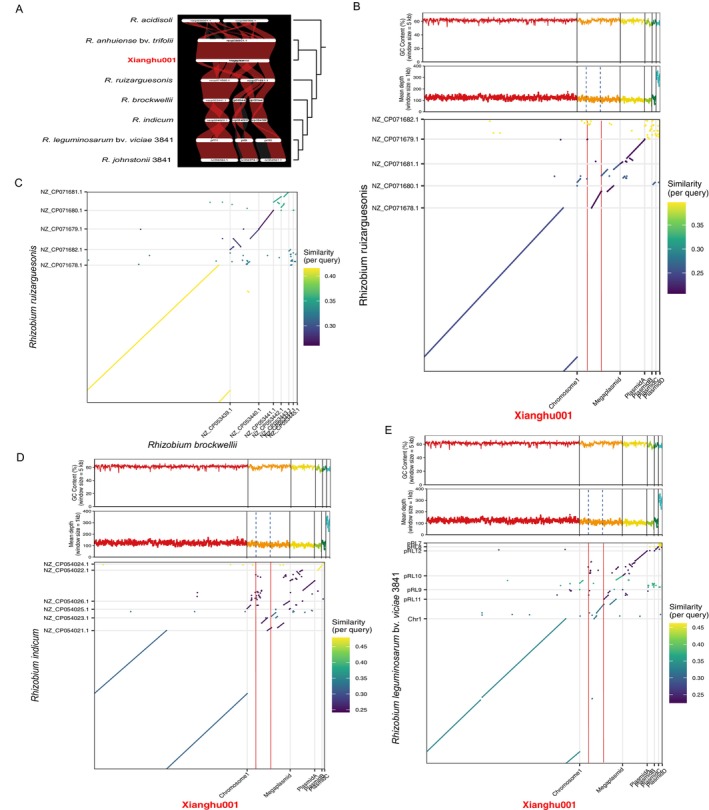
Inference of megaplasmid formation in *Rhizobium anhuiense*. (A) Megaplasmid synteny comparison of Xianghu001 and seven related representative *Rhizobium* strains. *Rhizobium leguminosarum* bv. *viciae* 3841 was renamed *Rhizobium johnstonii* 3841 in 2023 by Young et al. Red blocks represent homologous locally colinear blocks (LCBs) shared among plasmid. The right‐side dendrogram shows the phylogeny relationship based on whole genomic protein‐coding genes. (B) Pairwise genome comparison between Xianghu001 and *R. ruizarguesonis* strains including GC content profiles (top panel, 5 kb non‐overlap sliding window), sequencing depth distributions (middle panel, 1 kb non‐overlap sliding window) and nucleotide similarity dot‐plot (bottom panel). Similarity for per query is indicated by the colour gradient (see scale bar). Vertical dashed blue lines and red lines denote insertion sequences of Xianghu001. (C) Pairwise genome comparison between *R. ruizarguesonis* and *R. brockwellii*. (D) Pairwise genome comparison between Xianghu001 and *R. indicum*. The plasmidD of Xianghu001 is completey missing since there is no correspondence synteny blocks of between two genome assemblies. (E) Pairwise genome comparison between Xianghu001 and *R. leguminosarum* bv. *viciae* 3841.

Both chromosomal conservation and plasmid diversification were observed within *R. anhuiense* through genome synteny and phylogenetic analyses (Figure 2 and Table S9). The chromosome of *R. anhuiense* strain Xianghu001 exhibits high synteny with the chromosomes of nearly all other analysed *Rhizobium* species, indicating conserved gene order and structural stability across the genus. In contrast, the megaplasmid, identified as the largest replicon in Xianghu001, shows high synteny exclusively with *R. anhuiense* bv. *trifolii* (Figure 2). Comparative genomic analyses suggest that megaplasmid fission and fusion events have occurred in other *Rhizobium* species relative to *R. anhuiense*, contributing to plasmid diversification within the genus (Figures 2 and 3). Specifically, comparison among *R. anhuiense*, *R. ruizarguesonis* and *R. acidisoli* reveals that the megaplasmid in *R. anhuiense* corresponds to two separate plasmids in both *R. ruizarguesonis* and *R. acidisoli* (Figures 2 and 3). Given the phylogenetic position of *R. anhuiense*, the presence of a single fused megaplasmid appears to be unique to this lineage, with no similar plasmid fusion observed in either earlier‐ or later‐diverging lineages (Figures 2 and 3). To rule out the possibility of mis‐assembly, we performed independent de novo assemblies using Canu and Flye and compared them to the Unicycler assembly via whole‐genome alignment (Koren et al. 2017; Wick et al. 2017; Kolmogorov et al. 2019). The high alignment identity and one‐to‐one contig correspondence confirm the reproducibility of the fused megaplasmid structure, ruling out assembly artefacts and supporting biological authenticity (Figure S9).

In addition, a plasmid in *R. ruizarguesonis* appears to have undergone a fission event, giving rise to two separate plasmids in the lineage containing *R. brockwellii*, *R. indicum*, *R. leguminosarum* bv. *viciae* 3841 and *R. johnstonii* 3841 (Figures 2 and 3A). Notably, strain *R. leguminosarum* bv. *viciae* 3841 was reclassified as *Rhizobium johnstonii* 3841 in 2023 (Young et al. 2023); thus, the two labels in the figures refer to the same strain and genome. The smallest plasmids C and D in strain Xianghu001 display poor synteny with plasmids or chromosomal regions of other *Rhizobium* species, suggesting that these plasmids may have been acquired through horizontal gene transfer from distantly related taxa (Figure 2). Furthermore, insertion sequence (IS) element analysis of Xianghu001 revealed two ISs located within the megaplasmid, spanning 284,495–285,612 bp and 726,937–728,205 bp, respectively (Table S10). The latter is located at a predicted junction region, suggesting that IS‐mediated recombination may have facilitated plasmid fusion in this lineage (Figure 3B,D,E). These findings suggest that two independent IS‐mediated insertion events may have occurred in *R. anhuiense*, potentially contributing to the formation of the megaplasmid.

Together, these results highlight the dynamic and strain‐specific evolutionary history of Xianghu001's accessory genome, particularly its megaplasmid, in contrast to the highly conserved nature of its chromosome. The patterns of plasmid fusion, rearrangement and horizontal acquisition reflect the plasticity of the *Rhizobium* accessory genome and its potential adaptive significance in distinct rhizosphere environments.

### 3.3 Extensive Genetic Diversity Observed Within *Rhizobium anhuiense*

**FIGURE 4:**
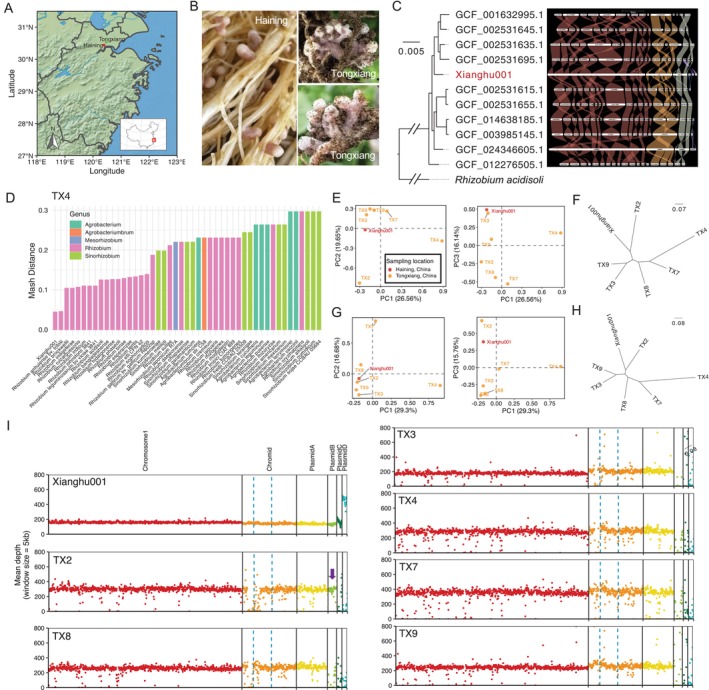
Genetic diversity among *Rhizobium anhuiense* strains. (A) Geographic locations of sample collection sites in Haining and Tongxiang, Zhejiang Province, China. (B) Root nodules formed on *Pisum sativum* plants in the field (Haining) and green house (Tongxiang). Xianghu001 isolated from Haining exhibits typical, individually formed nodules, whereas isolates from Tongxiang display clustered nodule morphology. (C) Phylogenetic tree based on genome alignments and chromosomal synteny analysis among *Rhizobium anhuiense* strains. Xianghu001 and its close relatives are highlighted in blue. Different coloured ribbons indicate conserved syntenic regions among genomes, with chromosomes scaled by gene rank order. (D) Representative Mash distance analysis of TX4 showing Xianghu001 was the closest relative among *Rhizobium* and related species. (E, F) Principal component analysis (PCA) of *R. anhuiense* strains based on (E) all SNPs and (F) only chromosomal SNPs. (G, H) Maximum‐likelihood trees based on (G) all SNPs and (H) only chromosomal SNPs, showing the genetic relationships among strains. Bootstrap support values (out of 100) are indicated at the nodes. (I) Read mapping depth across the genome of Xianghu001 for six TX strains. Each panel represents sequencing coverage (mean depth, 5‐kb non‐overlapping windows) across the chromosome, megaplasmid and four plasmids (A–D). Vertical dashed blue lines denote insertion sequences of Xianghu001. Purple arrow indicates confident detection of plasmid B in TX2.

We isolated six additional strains (designated by TX prefix) from root nodules of six *Pisum sativum* plants cultivated in Tongxiang, located approximately 10 km from Haining, the origin of strain Xianghu001 (Figure 4A). The genomes of these isolates were sequenced via next‐generation sequencing (NGS). Taxonomic identification was performed by blasting the short reads against the NCBI nt database, consistently identifying *R. anhuiense* as the top match for all six strains.

Interestingly, the morphology of the root nodules from Tongxiang (greenhouse) differed markedly from those observed in Haining (field) (Figure 4B and Figure S5). Nodules induced by strain Xianghu001 in Haining showed typical characteristics of *P. sativum* nodulation: numerous, well‐developed nodules that were individually distributed along the roots. In contrast, nodules associated with the TX strains from Tongxiang greenhouse generally formed only one or two nodules per plant, which frequently exhibited a branched or coralloid morphology rather than being uniformly distributed along the root system. However, when both the Haining and Tongxiang strains were incubated under nitrogen‐free conditions in the laboratory, almost no morphological differences were observed between them (Figure S6), suggesting that the observed differences in the field may be influenced by environmental factors rather than intrinsic genetic differences.

To investigate the genetic diversity of *Rhizobium anhuiense*, we collected the available genome assemblies, most of which were highly fragmented and lacked continuity, underscoring the utility of the complete, high‐quality Xianghu001 genome as a reference resource for *Rhizobium anhuiense*. Using an outgroup‐rooted whole‐genome phylogeny (rooted with *Rhizobium acidisoli*) with branch lengths, Xianghu001 showed closest affinity to a subset of *R. anhuiense* strains in the dataset (Figure 4C). Extensive chromosomal synteny was observed between Xianghu001 and other strains, whereas plasmid C and plasmid D exhibited limited syntenic conservation and fragmented alignment patterns (Figure 4C).

Pairwise Mash distance analysis among the six TX strains, Xianghu001 and other available *Rhizobium* genomes revealed that Xianghu001 was the closest relative to all TX strains (Figure 4D and Figure S7) (Ondov et al. 2016). Based on this, we selected Xianghu001 as the reference genome for subsequent analyses. A total of 194,101 SNPs were identified, among which 115,074 are chromosomally located. Principal component analysis (PCA) and phylogenetic reconstruction based on genome‐wide SNPs revealed substantial genetic diversity within *R. anhuiense*, with strains broadly distributed along the first three principal components (Figure 4E,F). Similar trends were observed when considering only chromosomal SNPs (Figure 4G,H). Notably, the genetic divergence between some TX strains exceeded that between some TX strains and Xianghu001, despite their close geographic proximity (Figure 4E–H). These findings reveal an unexpectedly high level of intra‐species genetic differentiation within *R. anhuiense*, suggesting complex evolutionary processes and possible subpopulation structuring, even among strains originating from nearby locations.

To further assess genomic variation, we examined sequencing depth profiles across the Xianghu001 genome for each TX strain (Figure 4I). All strains maintained high coverage across the chromosome and megaplasmid, indicating strong conservation of core genomic content. However, substantial variation was observed in the coverage of plasmids B, C and D, implying differential plasmid retention. Specifically, TX2 was presumed to retain plasmid B with sufficient read coverage, whereas TX3, TX4, TX7, TX8 and TX9 exhibited near‐zero coverage across multiple accessory plasmids, suggesting loss or significant rearrangements of these replicons. Some strains also showed abnormal depth peaks or drops, possibly reflecting structural variation or the presence of repetitive insertion sequences. Notably, plasmid B exhibited high synteny across 11 *R. anhuiense* genome assemblies, including Xianghu001 (Figure 4C).

Collectively, these results highlight the extensive genomic plasticity of *R. anhuiense*, particularly in its accessory genome. Plasmid gain, loss and recombination events appear to be major drivers of intraspecific diversity, potentially contributing to functional divergence and ecological adaptation in varied rhizosphere environments.

### 3.4 Plant Growth Promotion and Stress Tolerance of *Rhizobium anhuiense* Strain Xianghu001

**FIGURE 5:**
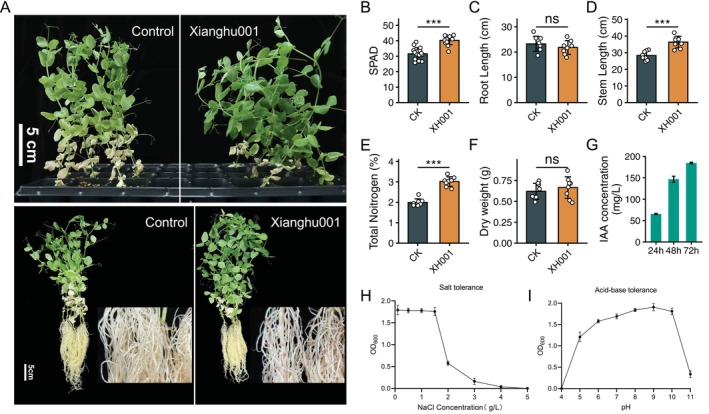
Plant growth promotion and stress tolerance of *Rhizobium anhuiense* strain Xianghu001. (A) Representative images of *Pisum sativum* plants inoculated with strain Xianghu001 versus control (uninoculated) after growth in sterile vermiculite with nitrogen‐free sterile water for 21 days after gemination. Upper panel shows whole plants; lower panel highlights root morphology and nodulation. (B–D) Quantification of SPAD (*n* = 16 per treatment) value (B), root length (*n* = 8 per treatment) (C) and stem length (*n* = 8 per treatment) (D) of plants with or without strain Xianghu001 inoculation. Data are shown as mean ± SD. For comparisons between CK and XH001 treatments, statistical significance was evaluated using a two‐tailed unpaired Student's *t*‐test. (E) Quantification of nitrogen content of plants with or without strain Xianghu001 inoculation (*n* = 8 per treatment). (F) Quantification of dry weight of plants with or without strain Xianghu001 inoculation (*n* = 8 per treatment). (G) IAA (indole‐3‐acetic acid) concentration in Xianghu001 culture supernatants at 24 h, 48 h and 72 h (*n* = 8). (H) Salt tolerance of Xianghu001, as measured by OD_600_ after growth under increasing NaCl concentrations after 48 h incubation with shaking (200 rpm) at 28°C (*n* = 3). Data are shown as mean ± SD. (I) Acid–base tolerance of Xianghu001, as assessed by OD_600_ at various pH conditions after 48 h incubation with shaking (200 rpm) at 28°C (*n* = 3). Data are shown as mean ± SD.

Rhizobia are known to promote legume growth by fixing nitrogen in the air. To evaluate the plant growth‐promoting potential of *R. anhuiense* strain Xianghu001, *P. sativum* seedlings were cultivated under nitrogen‐free conditions and inoculated with the strain. Compared to uninoculated controls, Xianghu001‐inoculated plants exhibited markedly enhanced shoot greenness (SPAD value), increased stem length and overall improved vigour 3 weeks after inoculation (Figure 5A,B–D and Table S11). Root morphology was notably altered, with the appearance of well‐developed nodules (Figure 5A), confirming successful symbiotic interaction. Chlorophyll quantification showed a 1.3‐fold increase in inoculated plants relative to controls (Figure 5B), consistent with visual phenotype. While root length was not significantly affected (Figure 5C), shoot height increased by 28.6% following rhizobial inoculation (Figure 5D). Furthermore, basal leaf senescence was notably delayed in treated plants: control plants showed complete senescence in the lowest four‐leaf pairs, whereas fewer than three pairs exhibited senescence in inoculated plants (Figure 5A). To verify biological nitrogen fixation, total plant nitrogen content was measured. Inoculated plants (XH001 is short for Xianghu001) accumulated 3.02% ± 0.08% nitrogen (mean ± SE), representing a statistically significant increase compared to the uninoculated control group (CK), which contained 1.99% ± 0.06% nitrogen (Figure 5E and Table S12). However, the dry weight has no significant difference between plants inoculated with and without the strain (Figure 5F).

Xianghu001 produced substantial levels of indole‐3‐acetic acid (IAA), a key phytohormone involved in root development and plant‐microbe signalling. IAA concentration in the culture supernatant increased steadily over time, reaching approximately 184 mg/L after 72 h of incubation (Figure 5G and Table S13) (Datta and Basu 1997, 2000). This sustained IAA production indicates active biosynthetic activity in the strain, which likely contributes to its observed plant growth‐promoting (PGP) effects (Mohite 2013). These findings highlight Xianghu001 as both an efficient nitrogen‐fixing symbiont and a free‐living PGP bacterium.

In terms of abiotic stress tolerance, Xianghu001 exhibited strong salt resistance up to 0.15% (w/v) NaCl, maintaining OD values above 1.7 (Figure 5H, Table 1 and Table S14). However, growth was sharply reduced at 0.2% and completely suppressed at higher salt concentrations, indicating a relatively narrow salt tolerance range (Figure 5H). In contrast, the strain displayed broad acid–base tolerance, with sustained growth across a wide pH range (5.0–10.0) (Figure 5I, Table 2 and Table S15). Optimal growth occurred under mildly alkaline conditions (pH 8.0–10.0), with OD_600_ values exceeding 1.8 (Figure 5I). No growth was observed at or below pH 4.0 and a pronounced decline in growth was detected at pH 11.0 (Figure 5I; Table 2).

**TABLE 1:** Salt tolerance performance of *R. anhuiense* strains.

NaCl (g/L)	Xianghu001	TX2	TX3	TX4	TX7	TX8	TX9
0.1	+	+	+	+	+	+	+
0.5	+	+	+	+	+	+	+
1.0	+	+	+	+	+	+	+
1.5	+	+	+	+	+	+	+
2.0	w	w	w	w	w	w	w
3.0	−	−	−	−	−	−	−
4.0	−	−	−	−	−	−	−
5.0	−	−	−	−	−	−	−

*Note:* +, positive; −, negative; w, weak growth.

**TABLE 2:** Acid–base tolerance performance of *R. anhuiense* strains.

pH	Xianghu001	TX2	TX3	TX4	TX7	TX8	TX9
4.0	−	−	−	−	−	−	−
5.0	+	+	+	+	+	+	+
6.0	+	+	+	+	+	+	+
7.0	+	+	+	+	+	+	+
8.0	+	+	+	+	+	+	+
9.0	+	+	+	+	+	+	+
10.0	+	+	+	+	+	+	+
11.0	w	w	−	w	w	+	w

*Note:* +, positive; −, negative; w, weak growth.

Comparative analysis of additional *R. anhuiense* strains isolated from *P. sativum* nodules in adjacent regions (Tongxiang, China) revealed largely similar stress response profiles (Tables 1 and 2). Notably, strain TX8 retained measurable growth at pH 11.0, suggesting the presence of strain‐specific genetic adaptations to extreme alkaline environments. These observations underscore both the conserved and divergent physiological traits within the *R. anhuiense* lineage, reflecting its ecological plasticity and potential for functional niche specialisation in diverse rhizosphere conditions.

## 4 Discussion

This study presents a comprehensive genomic and functional characterisation of *R. anhuiense*, revealing its unique plasmid architecture, significant intra‐species divergence and robust plant growth‐promoting traits. The complete genome and comparative analyses provide new insights into the evolutionary dynamics and adaptive strategies of *Rhizobium*. The dual capabilities of strain Xianghu001, efficient symbiotic nitrogen fixation and IAA‐mediated growth promotion, make it a promising candidate for biofertiliser development in sustainable agriculture. Further work exploring its gene regulatory networks, metabolomic profiles and field performance across soil types will enhance its applicability and facilitate precision microbiome engineering in legume cropping systems.

The generation of a complete, circularised genome for *Rhizobium* strain Xianghu001 represents a critical milestone in advancing our understanding of *R. anhuiense* genomics. By leveraging high‐coverage long‐read PacBio sequencing in combination with Illumina short‐read data, we achieved a contiguous and accurate assembly, comprising a chromosome, a megaplasmid and four additional plasmids. The assembly was validated by multiple tools, including BUSCO and CheckM, indicating full completeness and negligible contamination. This high‐quality reference genome provides a valuable platform for comparative genomic analyses and functional investigations of symbiotic and environmental adaptation in *Rhizobium*.

Plasmids are extrachromosomal genetic elements classified as mobile genetic elements (MGEs). Due to their ability to self‐replicate and transfer between bacterial cells, they play a crucial role in horizontal gene transfer. The genomic organisation of Xianghu001 revealed a distinctive plasmid architecture, particularly the presence of a large, single megaplasmid with high synteny to the megaplasmid of *R. anhuiens*e bv. *trifolii*. Comparative analysis with closely related strains such as *R. ruizarguesonis* and *R. acidisoli* showed that regions corresponding to the Xianghu001 megaplasmid are partitioned into multiple smaller plasmids in these species, indicating a plasmid fusion event unique to *R. anhuiense*. The identification of insertion sequences near the junction regions within the megaplasmid further suggests that IS‐mediated recombination could have facilitated this structural rearrangement. These findings highlight plasmid evolution as a key driver of diversification within *Rhizobium* species.

Despite its close geographic proximity to other isolates from Tongxiang (~10 km), Xianghu001 exhibits substantial genetic divergence. Genome‐wide SNP and PCA analyses revealed deep intra‐species diversity among *R. anhuiense* strains. Interestingly, the divergence among some TX strains exceeded their divergence from Xianghu001, suggesting potential local adaptation, niche partitioning, or cryptic population structure. The identification of strain‐specific plasmids, such as plasmids C and D in Xianghu001, with poor synteny to known *Rhizobium* replicons further points to the role of horizontal gene transfer in generating functional diversity within this species.

Auxin can promote nodule symbiosis in several ways. Infection thread is a structure in which rhizobia enter plant roots and its formation is regulated by auxin (Liu et al. 2015). Auxin at a certain concentration can induce nodule primordium formation and promote nodule initiation (Liu et al. 2018). The concentration of auxin secreted by Xianaghu 001 can reach 184 mg/L at 72 h of culture, which is higher compared to some other rhizobia reported in other study (Bianco et al. 2014; Lebrazi et al. 2020). It is well known that neither too high nor too low auxin concentration can promote plant growth and the amount of auxin secreted by the cultured rhizobia may be different from that during symbiosis. Therefore, it is worth further study to determine the concentration of auxin that can best promote the establishment of symbiosis. Considering the differences in sensitivity to phytohormones between species and even different varieties of plants, the concentration of auxin secreted by rhizobia may also be related to the adaptability of rhizobia to the host.

Xianghu001 exhibited moderate salt tolerance (up to 0.15% NaCl) and broad pH adaptability (5.0–10.0), with optimal growth in moderately alkaline environments. These traits likely confer competitive advantages in varied rhizosphere conditions, supporting persistence and function in different soils. In the future, the symbiotic efficiency of these rhizobia in stressed soil should be further explored to expand the application range of these rhizobia. The comparison with additional *R. anhuiense* strains from Tongxiang revealed both shared and strain‐specific responses to pH and salinity, particularly in TX8, which exhibited limited growth at pH 11.0. These findings underscore the physiological versatility within the species and suggest that genomic diversification underlies ecological fitness.

## 5 Conclusion

This study provides a complete genomic and functional characterisation of *Rhizobium anhuiense* Xianghu001, a pea‐nodulating symbiont isolated from Zhejiang Province. The circularised 7.36 Mb genome, comprising one chromosome, one megaplasmid and four additional plasmids, offers a high‐quality reference for understanding genome organisation and plasmid evolution in *R. anhuiense*. Comparative genomic analyses revealed a conserved chromosomal backbone but a highly dynamic accessory genome, including a putative lineage‐associated megaplasmid fusion and strain‐specific plasmids likely shaped by recombination and horizontal gene transfer.

Resequencing of six additional local isolates further uncovered substantial intraspecific diversity despite their close geographic origin. SNP‐based analyses and read‐depth profiling indicated that genomic divergence within *R. anhuiense* is driven not only by chromosomal variation but also by differential plasmid retention and accessory genome remodelling. These findings highlight the remarkable genomic plasticity of local rhizobial populations.

Functionally, Xianghu001 promoted pea growth under nitrogen‐deficient conditions, increasing chlorophyll content, shoot growth and total nitrogen accumulation. The strain also produced high levels of IAA and showed broad pH adaptability, with optimal growth under mildly alkaline conditions, although its salt tolerance was limited to low‐to‐moderate levels. Together, these traits support Xianghu001 as a promising candidate biofertiliser for pea production, particularly in neutral to mildly alkaline soils.

Overall, this work demonstrates that the agricultural potential of Xianghu001 is associated with both effective symbiotic performance and a dynamic accessory genome. Future studies should validate the mechanisms of megaplasmid fusion, clarify the functional roles of strain‐specific plasmids and evaluate the field stability and competitiveness of Xianghu001 across diverse soil environments.

## Author Contributions

**Jiashun Miao:** conceptualization, investigation, funding acquisition, writing – original draft, writing – review and editing, visualization, methodology, validation, software, formal analysis, project administration, data curation, supervision, resources. **Chi Zhang:** investigation, validation, writing – review and editing, formal analysis, methodology, writing – original draft. **Qifan Jiang:** investigation, validation, methodology, formal analysis, writing – review and editing, writing – original draft. **Zhangliang Yao:** investigation, writing – review and editing, methodology, validation, resources. **Kuirong Cao:** resources, investigation, writing – review and editing, validation, methodology. **Jie Chen:** resources, writing – review and editing, validation, investigation. **Hua Wang:** conceptualization, investigation, writing – original draft, writing – review and editing, visualization, validation, methodology, formal analysis, project administration, supervision, resources, data curation. **Na Liu:** conceptualization, investigation, funding acquisition, writing – original draft, writing – review and editing, methodology, validation, formal analysis, project administration, resources, supervision, data curation.

## Funding

This work was supported by the team development funding from Xianghu Laboratory, the Xiaoshan District Government and the Zhejiang Provincial Government and 2025 Special Cooperation Program between Xianghu Laboratory and Chinese Academy of Agricultural Science.

## Conflicts of Interest

The authors declare no conflicts of interest.

## Supporting information


**Figure S1:** Phylogenetic tree based on 16S *rRNA* gene sequences.
**Figure S2:** Phylogenetic tree based on 31 house‐keeping genes.
**Figure S3:** Average nucleotide identity (ANI)‐based heatmap of strain Xianghu001 (GL_1) and representative *Rhizobium* and related taxa.
**Figure S4:** Validation of the Xianghu001 genome assembly using multiple assemblers.
**Figure S5:** Nodule formation on *Pisum sativum* roots in field‐grown and greenhouse conditions.
**Figure S6:** Root morphology of *Pisum sativum* inoculated with different *Rhizobium anhuiense* strains.
**Figure S7:** Pairwise Mash distance profiles for strains TX2, TX3, TX7, TX8 and TX9 relative to *Rhizobium* and related genera.

**Table S1:** Summary statistics of PacBio long reads from strain Xianghu001.
**Table S2:** Read length distribution of PacBio long reads from strain Xianghu001.
**Table S3:** Summary statistics of Illumina short reads from strain Xianghu001.
**Table S4:** GC content and length statistics of the Xianghu001 genome assembly.
**Table S5:** BUSCO assessment of the Xianghu001 genome assembly.
**Table S6:** CheckM assessment of the Xianghu001 genome assembly.
**Table S7:** Genome coverage analysis of the Xianghu001 genome assembly.
**Table S8:** Summary statistics of repeat elements in the Xianghu001 genome assembly.
**Table S9:** Genome accession information for samples used in phylogenetic analysis.
**Table S10:** Insertion sequences identified in the Xianghu001 genome assembly.
**Table S11:** Root length, SPAD value and dry weight of pea plants under control and Xianghu001 inoculation treatments.
**Table S12:** Total nitrogen accumulation in pea plants under control and Xianghu001 inoculation treatments.
**Table S13:** IAA production by strain Xianghu001.
**Table S14:** OD600 values of strain Xianghu001 under different NaCl concentrations.
**Table S15:** OD600 values of strain Xianghu001 under different pH conditions.

## Data Availability

The raw PacBio reads and short reads of strain Xianghu001, and paired‐end short reads of six strains isolated from Tongxiang were deposited in NCBI under BioProject PRJNA1463013. The genome assembly, annotation files and other related resources for strain Xianghu001 are available on NCBI under the accession number JBYDFM000000000, Figshare (10.6084/m9.figshare.29828483) and our laboratory's FTP server at: http://t2tpgdb.xhlab.ac.cn/pisum/Xianghu001/.
