# Supplementary material for: Complete Genome of an Alkali‐Resistant Rhizobium anhuiense Symbiont of Pea Reveals Species‐Specific Plasmid Fusion and Genomic Plasticity
Source: Environ Microbiol Rep. 2026 May 25;18(3):e70366. doi: 10.1111/1758-2229.70366 (PMC13240570; doi:10.1111/1758-2229.70366)
Supplement: Supplementary file 1 — Figure S1: Phylogenetic tree based on 16S rRNA gene sequences. Figure S2: Phylogenetic tree based on 31 house‐keeping genes. Figure S3: Average nucleotide identity (ANI)‐based heatmap of strain Xianghu001 (GL_1) and representative Rhizobium and related taxa. Figure S4: Validation of the Xianghu001 genome assembly using multiple assemblers. Figure S5: Nodule formation on Pisum sativum roots in field‐grown and greenhouse conditions. Figure S6: Root morphology of Pisum sativum inoculated with different Rhizobium anhuiense strains. Figure S7: Pairwise Mash distance profiles for strains TX2, TX3, TX7, TX8 and TX9 relative to Rhizobium and related genera. [file EMI4-18-e70366-s001.docx]

**Supplementary Materials**

**Supplementary Figures**

Supplementary Figure 1. Phylogenetic tree based on 16S *rRNA* gene sequences

Supplementary Figure 2. Phylogenetic tree based on 31 house-keeping genes

Supplementary Figure 3. Average nucleotide identity (ANI)-based heatmap of strain Xianghu001 (GL_1) and representative *Rhizobium* and related taxa

Supplementary Figure 4. Validation of the Xianghu001 genome assembly using multiple assemblers

Supplementary Figure 5. Nodule formation on *Pisum sativum* roots in field-grown and greenhouse conditions

Supplementary Figure 6. Root morphology of *Pisum sativum* inoculated with different *Rhizobium anhuiense* strains

Supplementary Figure 7. Pairwise Mash distance profiles for strains TX2, TX3, TX7, TX8, and TX9 relative to *Rhizobium* and related genera

**Supplementary Tables**

Supplementary Table 1. Summary statistics of PacBio long reads from strain Xianghu001

Supplementary Table 2. Read length distribution of PacBio long reads from strain Xianghu001

Supplementary Table 3. Summary statistics of Illumina short reads from strain Xianghu001

Supplementary Table 4. GC content and length statistics of the Xianghu001 genome assembly

Supplementary Table 5. BUSCO assessment of the Xianghu001 genome assembly

Supplementary Table 6. CheckM assessment of the Xianghu001 genome assembly

Supplementary Table 7. Genome coverage analysis of the Xianghu001 genome assembly

Supplementary Table 8. Summary statistics of repeat elements in the Xianghu001 genome assembly

Supplementary Table 9. Genome accession information for samples used in phylogenetic analysis

Supplementary Table 10. Insertion sequences identified in the Xianghu001 genome assembly

Supplementary Table 11. Root length, SPAD value, and dry weight of pea plants under control and Xianghu001 inoculation treatments

Supplementary Table 12. Total nitrogen accumulation in pea plants under control and Xianghu001 inoculation treatments

Supplementary Table 13. IAA production by strain Xianghu001

Supplementary Table 14. OD600 values of strain Xianghu001 under different NaCl concentrations

Supplementary Table 15. OD600 values of strain Xianghu001 under different pH conditions


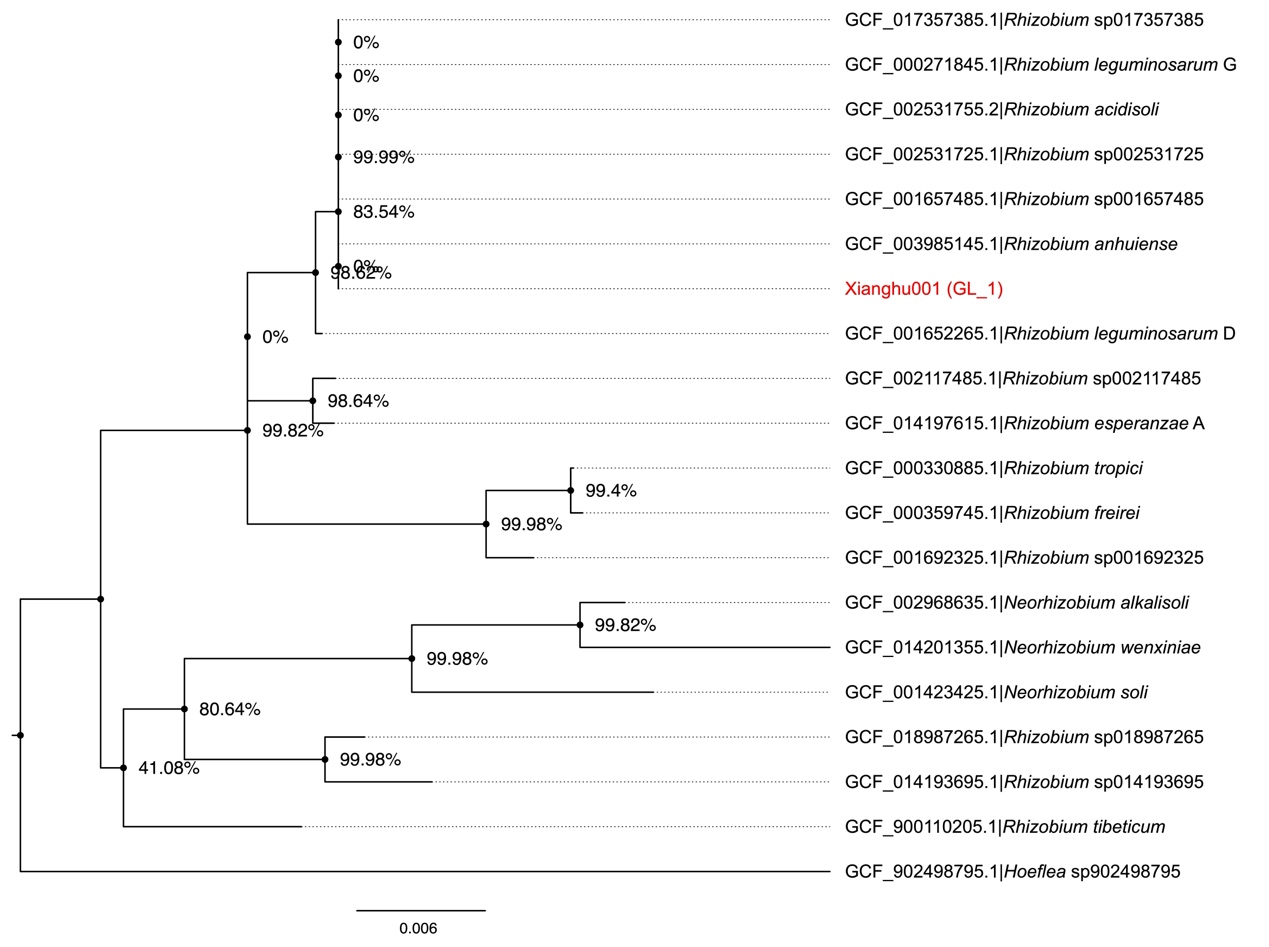


**Supplementary Figure 1. Phylogenetic tree based on 16S *rRNA* gene sequences**

A neighbor-joining tree was constructed using the 16S *rRNA* gene sequences of strain Xianghu001 (GL_1) and representative species within the *Rhizobiaceae* family. The analysis was performed using MEGA version 6.0 with default parameters. Bootstrap support values were displayed at the internal nodes. Strain Xianghu001 (highlighted in red) clustered within the *Rhizobium anhuiense* clade, supporting its assignment to the *R. anhuiense* species. Species names and corresponding NCBI GenBank assembly accession numbers are displayed on separate lines.


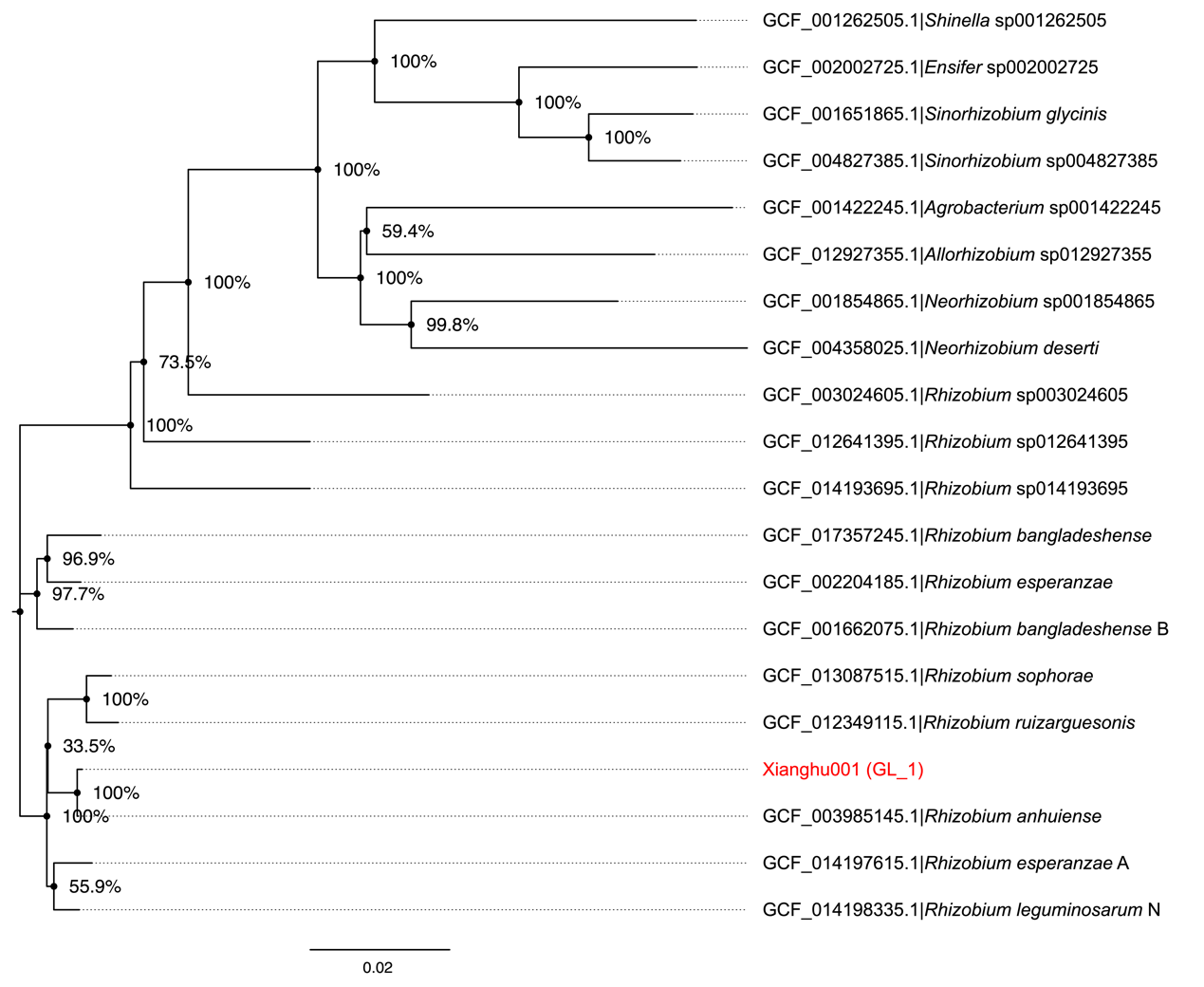


**Supplementary Figure 2. Phylogenetic tree based on 31 house-keeping genes**

MEGA (version 6.0) software was employed to generate a neighbor-joining tree with 31 house-keeping genes (*dnaG*, *frr*, *infC*, *nusA*, *pgk*, *pyrG*, *rplA*, *rplB*, *rplC*, *rplD*, *rplE*, *rplF*, *rplK*, *rplL*, *rplM*, *rplN*, *rplP*, *rplS*, *rplT*, *rpmA*, *rpoB*, *rpsB*, *rpsC*, *rpsE*, *rpsI*, *rpsJ*, *rpsK*, *rpsM*, rpsS, *smpB*, *tsf*). Bootstrap support values were displayed at the internal nodes. Strain Xianghu001 (GL_1, highlighted in red) clusters closely with *Rhizobium anhuiense*, supporting its taxonomic assignment to the *R. anhuiense* lineage. For each taxon, the species name and the corresponding NCBI GenBank assembly accession number are shown on separate lines.


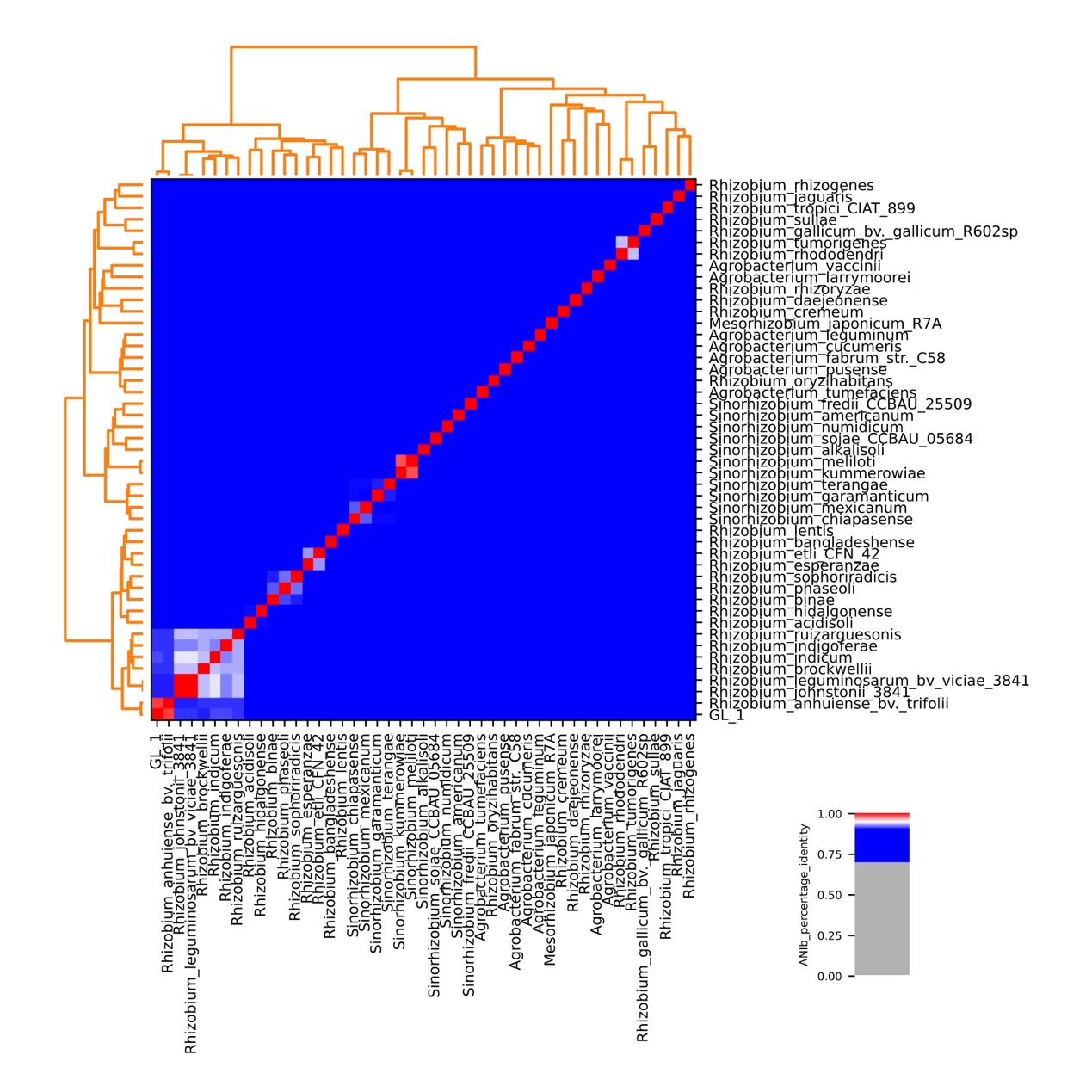


**Supplementary Figure 3. Average nucleotide identity (ANI)-based heatmap of strain Xianghu001 (GL_1) and representative *Rhizobium* and related taxa**

ANI values were calculated between strain Xianghu001 (GL_1) and other representative strains from the *Rhizobiaceae* family and closely related genera. Pairwise comparisons were performed using whole-genome nucleotide sequences. The heatmap displays the pairwise ANI values, with color intensity representing the degree of identity (from 0 to 1.0, as shown in the scale bar). Hierarchical clustering based on ANI similarity is shown along both axes. Strain Xianghu001 (GL_1) clusters tightly with *Rhizobium anhuiense*, confirming its taxonomic affiliation. Species names are indicated on both axes for cross-reference.

**Supplementary Figure 4. Validation of the Xianghu001 genome assembly using multiple assemblers**

(A-B) Whole-genome alignment dotplots comparing the Unicycler assembly of strain Xianghu001 to assemblies generated by (A) Canu and (B) Flye. Each plot shows sequence similarity across chromosome or replicons, with the color gradient indicating alignment identity (per query). The Unicycler assembly is used as the reference (x-axis), and the comparison assemblies are on the y-axis. High collinearity and alignment identity (yellow diagonal lines) indicate consistent structural features across assemblers and support the robustness of the Unicycler-based genome structure. These results confirm the presence of a fused megaplasmid in Xianghu001, ruling out assembler-specific assembly artifacts.

**Supplementary Figure 5. Nodule formation on *Pisum sativum* roots in field-grown and greenhouse conditions**

(A-B) Abundant root nodules formed on *Pisum sativum* roots under natural field conditions, showing pinkish interior indicative of active nitrogen fixation. (C-E) Close-up images of individual nodules (circled in red) on *Pisum sativum* roots under greenhouse conditions. The nodules exhibit typical pink coloration, suggesting functional leghemoglobin and active nitrogen fixation. However, only one root nodule was found in single *Pisum sativum* plant.

**Supplementary** **Figure 6. Root morphology of *Pisum sativum* inoculated with different *Rhizobium anhuiense* strains**

(A-C) Xianghu001 strain from Haining. (D-F) TX2 strain from Tongxiang. Images show individual root systems with nodules formed under identical growth conditions.

**Supplementary Figure S7. Pairwise Mash distance profiles for strains TX2, TX3, TX7, TX8, and TX9 relative to *Rhizobium* and related genera**

In each panel, the query strain (indicated in the upper-left corner) was compared against a set of reference genomes, and bars represent pairwise Mash distances (sorted in ascending order). Bar colors denote the genus of each reference genome. In all panels, Xianghu001 shows the lowest MASH distance and is therefore the closest relative to each query strain.
